# Potential Impact of Biologic Use on Asthma‐Related Hospitalization: An Epidemiological Study in Hong Kong

**DOI:** 10.1155/carj/5535762

**Published:** 2026-07-15

**Authors:** Wang Chun Kwok, Chung Ki Tsui, Ting Fung Ma, Shuk Man Ngai, Lu Zhou, Isaac Sze Him Leung, Man Fung Tsoi, Raymond Yau Hang Leung, Ru Zhang, James Chung Man Ho

**Affiliations:** ^1^ Department of Medicine, Queen Mary Hospital, The University of Hong Kong, 102 Pokfulam Road, Pokfulam, Hong Kong SAR, China, hku.hk; ^2^ Department of Statistics, University of South Carolina, Columbia, South Carolina, USA, sc.edu; ^3^ Department of Statistics, The Chinese University of Hong Kong, Shatin, New Territories Hong Kong SAR, China, cuhk.edu.hk

**Keywords:** asthma, asthma exacerbation, biologics

## Abstract

**Background:**

While the individual effects of biologics in asthma are well demonstrated, the potential impact of the use of biologics on overall asthma‐related hospitalization at a population‐based level has not been reported.

**Methods:**

A territory‐wide study conducted in Hong Kong, involving all adult patients with asthma managed in Hospital Authority. The primary outcome was the change in asthma‐related hospitalization from 2014 to 2023, correlating with the number of biologic use among patients with asthma and adjusted for Air Quality Health Index and influenza case number.

**Results:**

The weekly asthma‐related hospitalization was 96 episodes at the start of the study period and 78 episodes at the end of the study period. There was a significant reduction of asthma‐related hospitalization associated with biologic usage, giving the estimated risk ratio of 0.994 (95% CI = 0.991–0.996, *p* value < 0.05). The estimated break‐point for the change in asthma‐related hospitalization was at Week 169 (95% CI = 130.3–207.7), which was the week of 19/3/2017 to 25/3/2017. The slopes before and after the break‐point were 0.022 and −0.191 with the difference in the two slope estimates being significant (*p* < 0.001) according to the Davies test. The significant difference in the slope before and after the break‐point suggested that beyond Week 169, there was a trend of significant reduction in asthma‐related hospitalization.

**Conclusion:**

We observed a reduction in asthma‐related hospitalizations throughout the study period with a potential temporal association with the increase in biologic prescription. While biologics provided benefits in individual level, the possible benefits in a population‐based level could be related to a more comprehensive care in severe asthma patients from the introduction of biologics for severe asthma.

**Summary**
• New knowledge added by this study
◦ A possible temporal association between the use of biologics for asthma and reduction in asthma‐related hospitalizations was observed.
◦ There was a major change in trend of asthma‐related hospitalizations with a continuous drop in weekly asthma‐related hospitalizations, which almost matched with the timing of launch of mepolizumab, the most commonly prescribed asthma biologic in Hong Kong.
• Implications for clinical practice or policy
◦ The benefits from the introduction of biologics for asthma through a more comprehensive care of severe asthma patients and the use of biologics in a population level were postulated.

## 1. Introduction

Severe asthma is defined by the requirement of high‐dose inhaled corticosteroid plus a second controller and/or systemic corticosteroid for 50 percent or more of the time in a year to prevent asthma from becoming uncontrolled or that which remains uncontrolled despite this therapy [1, 2]. Severe asthma remains a major healthcare problem and is associated with significant morbidity and mortality [3–6].

The development of biologics has revolutionized the treatment paradigm in severe asthma [7–12]. Initially, biologics were exclusively developed for those with Th2 asthma [13], while anti–thymic stromal lymphopoietin (TSLP), namely, tezepelumab, also worked for non‐Th2 asthma [14]. Although the biologics for severe asthma have not been compared in head‐to‐head trials, indirect comparisons found similar improvements in terms of exacerbation rates and asthma control [15]. While the individual effect of biologics in asthma is well demonstrated in clinical trials and real‐world studies [16], whether the use of biologics will have an impact on overall asthma‐related hospitalization in population‐based level has not been reported. Although there is no doubt about the effectiveness of biologics in asthma treatment, the costs of asthma biologics are also a concern. Whether biologics in severe asthma can bring about benefits in population‐based level will be meaningful for policymakers to assess the impact of biologic introduction and prescription. If there is pronounced benefit in population‐based level, a more liberal use of biologics should be evaluated.

To prescribe biologics for severe asthma, there is a need of phenotyping in order to choose the appropriate biologics. Given the high costs of biologics, various countries and places also develop guidelines and protocols on the use of biologics, to limit the use of biologics among patients who fulfill certain clinical criteria. As such, for patients who are considered for biologic use, there is need for proper patient evaluation on the exacerbation history, lung function and phenotype, and assessment by accredited specialists. Whether these will translate into better patient outcomes from a population‐based level is lacking.

In view of the lack of data on the potential association of biologic use at a population‐based level, we conducted this epidemiological study to investigate the impact of biologic use in asthma‐related hospitalization.

## 2. Materials and Methods

This is a territory‐wide epidemiological study conducted in Hong Kong. Adult patients with asthma managed in Hong Kong Hospital Authority (HKHA) from 2014 to 2023 were included.

This study utilized electronic health records from the Clinical Data Analysis and Reporting System (CDARS) managed by the HKHA. HKHA is a public healthcare service provider that manages 43 hospitals and institutions and 122 outpatient clinics, covering more than 90% of the Hong Kong population since 1993 [17]. The CDARS captures medical information including diagnosis, drug prescription details, demographics, admissions, medical procedures, and laboratory results. The diagnostic code of asthma (493 in International Classification of Diseases, Ninth Revision) in CDARS was validated with a positive predictive value (PPV) of 85.0% (95% CI: 80.1–89.9%). This is based on the following criteria: A potential asthma case was considered as true positive if the specialist regarded the patient to have definite asthma: if the patient has history of intermittent symptoms typical of asthma (wheeze, shortness of breath, chest tightness, cough, that vary over time and intensity) and relevant medication record (prescription of bronchodilator, inhaled corticosteroid, leukotriene receptor antagonists, theophylline), supported by variable expiratory airflow limitation demonstrated by spirometry in doubtful cases, with alternative diagnoses to account for the clinial presentation being excluded [18].

The inclusion criteria included adult patients (age at or above 18 years old) with physician‐labeled asthma at or before 31st December 2022. Patients with asthma were identified by International Classification of Diseases 9th Revision (ICD‐9) code of 493 (493.0, 493.1, 493.2, and 493.9) from CDARS. Patients with coexisting ICD‐9 code of 496, which suggested coexisting diagnosis of chronic obstructive pulmonary disease, were excluded. Patients who were lost to follow up were also excluded. The demographics (age and gender) and clinical characteristics (onset of asthma, Charlson Comorbidity Index [CCI], baseline blood eosinophil count [BEC], medications for asthma, and the severity of asthma based on GINA steps as in Version 2024) were retrieved from CDARS. The number of asthma‐related hospitalizations was retrieved from CDARS, from 1st January 2014 to 31st December 2023.

At the time of data cutoff, there were 4 biologics available in Hong Kong (omalizumab, dupilumab, benralizumab, and mepolizumab). All of them are covered by the Samaritan Fund, for patients who fulfilled the clinical and financial criteria. The clinical criteria for the Samaritan Fund application, date of registration in Hong Kong, and also the date of inclusion in the Samaritan Fund are presented in Supporting Table 1.

Influenza incidence was collated from territory‐wide laboratory surveillance data from the Centre for Health Protection of Hong Kong [19]. Air quality was measured by the Air Quality Health Index (AQHI), which was obtained from the Hong Kong Environmental Protection Department. AQHI assessed the cumulative health risk attributable to 3‐hour moving average concentrations of four air pollutants (ozone, nitrogen dioxide, sulfur dioxide, and particulate matter [PM_2.5_/PM_10_]) [20]. AQHI was reported on a scale of 1–10 and 10+ and was grouped into five different health risk categories: low (1–3), moderate (4–6), high (7), very high (8–10), and serious (10+). The AQHI was reported hourly in 13 stations located in different areas of Hong Kong. The total number of hours with the AQHI recorded as high to serious grades was summed up and expressed as a percentage of the total number of hours collected in a month.

The primary outcome was the change in asthma‐related hospitalizations from 2014 to 2023, correlating with the number of biologic use among patients with asthma. The study was approved by the Institutional Review Board of the University of Hong Kong and Hospital Authority Hong Kong West Cluster (UW 23‐510).

### 2.1. Statistical Analysis

Numerical data based on demographic features and biomarker results were presented as mean and standard deviation (SD) as most of these were normally distributed variables. Median and interquartile range (IQR) were used to present numerical data that did not follow normal distribution.

For comparison of the difference in asthma‐related hospitalization counts between weeks, log‐linear modeling was used to model count, and “weeks” was treated as a covariate in the model. An offset term is also included in the model to adjust for the varying total number of patients during the observation time. Segmented regression model [21, 22] was performed for identifying the potential and unknown break‐point of the trend in the number of hospitalization over time. Two log‐linear models were fitted for the trends before and after the break‐point, which had separate intercepts and regression coefficients and captured the heterogeneity of the data. Follow‐up analysis, such as the statistical significance of the change in slope and existence of break‐point, was conducted. The confidence interval of the break‐point was also estimated to quantify the variability of the estimated break‐point via the delta method. The average yearly asthma‐related hospitalizations and the SD for asthma from January 2014 to December 2023 were compared. Two‐sided tests were conducted for general difference, and we set 5% as the significance level in the analysis. The weekly influenza case number and AQHI were included as covariates in the multivariate analysis, to be adjusted as confounders for changes in asthma‐related hospitalization number. Statistical significance was determined at the level of *p* < 0.05. All statistical analyses were performed using R Version 4.2.2 (2022‐10‐31).

## 3. Results

There were a total of 101,521 adult patients with asthma included in this study. From January 2014 to December 2023, there were 59,370 asthma‐related hospitalizations. The weekly number of asthma‐related hospitalizations was 96 episodes at the start and 78 episodes at the end of the study period. The mean age of the patients was 57.5 ± 20.0 with 43,926 (43.3%) being male. The median CCI was 1 [0–3]. The median BEC was 200 [100–360] cells/μL. The median eosinophil percentage was 2.29 [1.0–4.5] %. 35.3% of the patients had eosinophilic asthma, as defined by BEC ≥ 300 cells/μL [23]. The results are summarized in Table 1. The baseline characteristics of the patients were not adjusted in the analyses. The weekly influenza case numbers and AQHI are included in Supporting Figures S1 and S2.

**TABLE 1:** Baseline demographics of the included patients.

Number of subjects	101,521
Age (years), mean ± SD	57.47 ± 20.02
Male (%)	43,926 (43.3%)
Age of asthma diagnosis (years), mean ± SD	45.12 ± 19.95
CCI, median (IQR)	1 (0–3)
Baseline blood eosinophil count (BEC), cells/μL, median (IQR)	200 (100–360)
Baseline blood eosinophil percentage, median (IQR)	2.29 (1.0–4.5)
Patients with ≥ 150 cells/μL (%)	57.4%
Patients with BEC ≥ 150 cells/μL (%)	35.3%

*Note:* GINA, Global Initiative for Asthma.

Abbreviations: BEC, blood eosinophil count; CCI, Charlson Comorbidity Index; IQR, interquartile range; SD, standard deviation.

**FIGURE 1:**
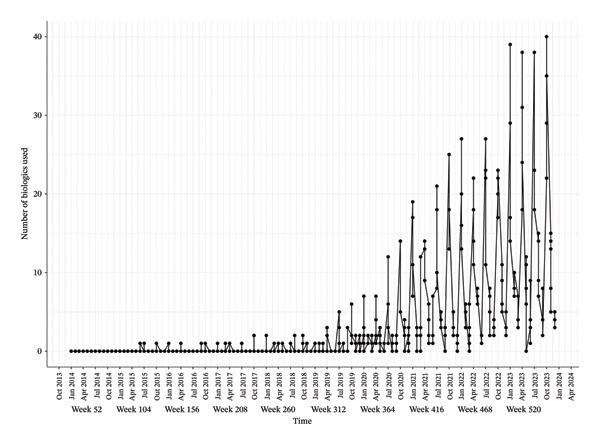
Number of biologics prescribed for asthma patients from 2014 to 2023. *Y*‐axis: number of biologics prescribed. *X*‐axis: time. This figure presented the weekly number of biologics prescribed from 2014 to 2023.

There were 268 patients treated with biologics in this cohort, with mepolizumab being the most commonly used one, followed by benralizumab, dupilumab, and omalizumab. 115, 57, 49, and 47 patients were prescribed with mepolizumab, benralizumab, dupilumab, and omalizumab, respectively. The trend of biologic prescription is illustrated in Figure 1.

**FIGURE 2:**
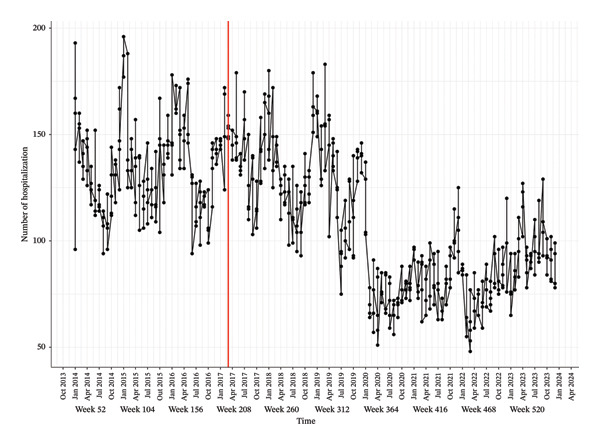
Number of asthma‐related hospitalizations from 2014 to 2023. *Y*‐axis: number of asthma‐related hospitalizations. *X*‐axis: time. This figure presented the weekly number of asthma‐related hospitalizations with the break‐point at Week 169 (19/3/2017 to 25/3/2017) illustrated with a red vertical line.

There was a significant reduction of asthma‐related hospitalization associated with biologic usage with the estimated risk ratio of 0.994 (95% CI = 0.991–0.996, *p* value < 0.05). The results are illustrated in Table 2 and Figure 2.

**TABLE 2:** Annual number of asthma‐related hospitalization from 2014 to 2023.

		Year 2014	Year 2015	Year 2016	Year 2017	Year 2018	Year 2019	Year 2020	Year 2021	Year 2022	Year 2023
Total number (counts) of asthma‐related hospitalization in the year		4823	4866	4967	5160	4778	4721	2839	2932	2700	3002

Total number of patients in each year (including both groups)		101,521	101,229	100,736	100,220	99,730	99,144	98,543	97,796	96,784	95,864

Biologic group (among 101,521 asthma patients)	Number of patients in the subgroup in each year	0	1	2	3	5	22	63	113	154	223
	Total number (counts) of asthma‐related hospitalization in the year	0	1	2	3	5	20	56	93	112	104

No biologic group (among 101,521 asthma patients)	Number of patients in the subgroup in each year	101,521	100,958	100,734	100,217	99,725	99,122	95,780	97,683	96,630	95,641
	Total number (counts) of asthma‐related hospitalization in the year	4823	4865	4965	5157	4773	4701	2783	2839	2588	2898

**FIGURE 3:**
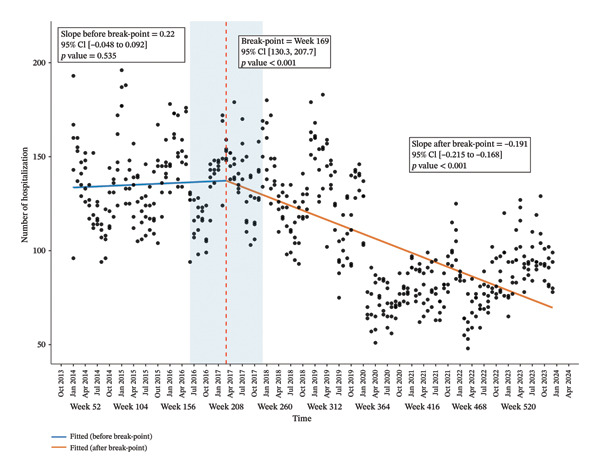
Segmented regression model on break‐point of change in asthma‐related hospitalizations from 2014 to 2023. *Y*‐axis: number of asthma‐related hospitalizations. *X*‐axis: time. The slopes before and after break‐point were 0.022 and −0.191, respectively. The *p* value for the difference in the slope before and after the break‐point (Week 169 [19/3/2017 to 25/3/2017]) was < 0.001, suggesting there was significant reduction in asthma‐related hospitalization beyond this break‐point.

Break‐point analysis was performed using the R package segmented [24] to determine the period in which there was a major change in asthma‐related hospitalization. The estimated break‐point was at Week 169 (95% CI = 130.3–207.7), which was the week of 19/3/2017 to 25/3/2017. The slope before the break‐point was 0.022 (*p* = 0.535), while it was −0.191 (*p* < 0.001) after the break‐point (Table 3). These results suggest that before the break‐point, there was no obvious trend in the asthma‐related hospitalization with a nonsignificant *p* value, while after the break‐point, there was a significant reduction in asthma‐related hospitalization. The difference in the two slope estimates was significant (*p* < 0.001) according to the Davies test with a slope of −0.214 (Figure 3). This suggested that beyond the week of 19/3/2017 to 25/3/2017, there was a significant reduction in asthma‐related hospitalization.

**TABLE 3:** Results from segmented regression.

Segment	Estimate	Standard error	*T*‐statistic	95% CI	*p* value
Slope before the break‐point	0.022	0.036	0.621	–0.048 to 0.092	0.535
Slope after the break‐point	–0.191	0.012	–16.268	–0.215 to −0.168	< 0.001
Difference in slope	–0.214	0.037	–5.705	–0.287 to −0.140	< 0.001

Coincidentally, the estimated break‐point was right before the introduction of mepolizumab, one of the first most commonly used asthma biologics for Th2 asthma in Hong Kong (Supporting Table 1), in line with the potential impact due to biologic prescription.

## 4. Discussion

In this epidemiological study, the potential temporal association between the use of biologics for asthma and a reduction in asthma‐related hospitalizations was observed. While the benefits from individual biologics were demonstrated from randomized controlled trials and real‐world studies, whether the availability of biologics, including the access and prescription of biologics, has implications in population level also worth assessing. In our study, we demonstrated that in 2017 quarter 1 (week of 19/3/2017 to 25/3/2017), there was a major change in trend of asthma‐related hospitalizations with a continuous drop in weekly asthma‐related hospitalizations. The timing of this change was close to the timing of launch of mepolizumab, the most commonly prescribed asthma biologic in Hong Kong. The results in this study may suggest population‐level temporal association between biologic use and asthma‐related hospitalizations. The association could result from increase in awareness of severe asthma and its treatment, better patient care in preparation of biologic prescription, and biologic use for severe asthma.

Our study noted a temporal association between the number of biologic use and the number of asthma‐related hospitalizations in Hong Kong from a population‐based level. With the introduction of mepolizumab, benralizumab, and dupilumab since 2017 and the incorporation of all 4 biologics (omalizumab, mepolizumab, benralizumab, and dupilumab) in the Samaritan Fund, there has been a sustained and marked increase in biologic prescription since then. Almost at the same time, a reduction of asthma‐related hospitalizations was noted, starting from the week of 19/3/2017 to 25/3/2017 and persisting till the end of the study period. From the epidemiological data, the break‐point suggesting the reduction of asthma‐related hospitalizations was close to the timing of registration of mepolizumab, the most commonly used asthma biologic in Hong Kong. Subsequently, there was an increase in availability and prescription of biologics, as well as the introduction of these biologics in the Samaritan Fund with financial subsidy provided to eligible patients. This resulted in increase in biologic use among severe asthma patients. Temporally, there was reduction in asthma‐related hospitalizations beyond the break‐point. While the Samaritan Fund is a public funding scheme applicable to the whole population, there were patients entitled to free asthma biologics in Hong Kong under civil service terms before the inclusion in the Samaritan Fund. These patients include civil servants, retired civil servants, pensioners, dependents of civil servants, and staff of HKHA and their spouse who were also eligible for free biologic use without the need of support from the Samaritan Fund. Currently, there are more than 170,000 civil servants in Hong Kong and more than 90,000 employees of HKHA, respectively. They could use these biologics for free once they were registered in Hong Kong and financial assessment was not needed. The access to biologics was also proven to be associated with improvement in asthma‐related outcomes in other countries. An American study suggested that insurance coverage is associated with improved asthma control for adults aged 18 to 64 years from households with low socioeconomic status [25]. Another study also suggested that out‐of‐pocket medication costs for asthma in pediatric population being associated with asthma exacerbation risks. For each percentage point increase in the proportion of income devoted to out‐of‐pocket payment for asthma medication, the rate of exacerbation will be increased by 14% [26]. These suggest that access to treatment and the out‐of‐pocket payment are factors related to asthma‐related outcomes. The availability of funding scheme, such as the Samaritan Fund in Hong Kong, could at least partly overcome the hurdle in appropriate biologic treatment for severe asthma.

While the drastic drop in 2020 might be related to the coronavirus disease 2019 (COVID‐19) outbreak [27, 28] with stringent infection control measures, the continuous low number of asthma‐related hospitalizations could not be explained by the infection control measures related to COVID‐19. There was gradual loosening of various infection control measures since late 2022. There was also the fifth wave COVID‐19 outbreak in early 2022 with a large population being infected. This led to worsening of asthma control as our group has demonstrated previously [29]. Despite the negative impact of COVID‐19 infection, there was continuous reduction of asthma‐related hospitalizations from 2020 to 2023, at the time when the COVID‐19 pandemic was dying downwith the World Health Organization (WHO) eventually declared the end of the pandemic phase of COVID‐19 on May 5, 2023. This reduction could be temporally related to the continuous increase in biologic prescription over that period.

Patients prescribed with biologics will likely benefit from exacerbation reduction. The availability of biologics to the pateints will be enhanced by Samaritan Fund coverage. But in the process of patient selection and assessment for biologic prescription, it also involves asthma phenotyping and detailed assessment by specialists. To screen for biologic suitability, clinical evaluation with phenotyping is a must, with blood for BEC, IgE level, and lung function test. Patients will also be assessed by respiratory specialists more frequently, as respiratory specialists’ assessment and endorsement are prerequisites for Samaritan Fund applications for biologics. This could also bring about better asthma control with reduced exacerbation, which is a phenomenon also observed in clinical trials with biologics, with a modest clinical benefit seen in the placebo group. Nonetheless, biologics should be prescribed in the patients with severe eosinophilic asthma if indicated, as the benefits are clearly demonstrated in multiple clinical studies. For those who may potentially be suitable for biologics yet eventually failed, more intensive monitoring with reevaluation is also warranted, especially when the indications of biologics are extending beyond severe Th2 asthma, with the introduction of tezepelumab.

In our study, we also assessed the number of influenza cases and air quality by AQHI, which are important factors that will affect asthma‐related hospitalizations. After adjusting for these two important factors, we demonstrated the inverse relationship between the decrease in asthma‐related hospitalizations and the increase in use of biologics. This is one of the strengths in this study.

There are a few limitations in our study. First, the majority of the patients included are Chinese. Nonetheless, the clinical features of asthma and the benefits of biologics are consistent over different ethnic groups. Second, the effect of individual biologics is not compared. But it is not the primary objective of the study, and the relatively small number of patients precludes comparison of the 4 biologics for asthma. Third, time series models and seasonality are not fully considered in the analysis mainly due to the computational challenges when the response is not continuous and the relatively small sample size. In general, it could be addressed by genetic change point detection methods, such as Ma and Yau et al. [30], for approximated inference using much higher computational cost. We believe that it is reasonable to assume that the dependence over time could be captured by the regression line. The study period also overlaps with the COVID‐19 pandemic, in which the asthma‐related hospitalizations were low partly due to lockdown [27, 28]. This could explain the low asthma‐related hospitalization numbers from 2020 to 2022. But the social distancing and other related public health policies were lowered and then removed from mid‐2022. Hence, the low asthma‐related hospitalization number in 2023 could not be attributed to COVID‐19. Also, the drop in asthma‐related hospitalization number started back in 2017, which was also suggested by the break‐point analysis.

## 5. Conclusion

We observed a reduction in asthma‐related hospitalizations throughout the study period with a potential temporal association with the increase in biologic prescription. While biologics provided benefits in individual level, the possible benefits in a population‐based level could be related to a more comprehensive care in severe asthma patients from the introduction of biologics for severe asthma.

## Author Contributions

W.C.K. and T.F.M. were involved in study concept and design; C.K.T., R.Y.H.L., and S.M.N. were involved in acquisition of data; L.Z., W.C.K., T.F.M., I.S.H.L., R.Z., and M.F.T. were involved in analysis and interpretation of data; W.C.K., T.F.M., C.K.T., and I.S.H.L. were involved in drafting of the article; and J.C.M.H. was involved in critical revision for important intellectual content. All authors had full access to the data, contributed to the study, and take responsibility for its accuracy and integrity. All authors contributed equally to this work.

## Funding

This study was partially sponsored by GlaxoSmithKline Hong Kong.

## Disclosure

All authors have given final approval of the version to be published, have agreed on the journal to which the article has been submitted, and agree to be accountable for all aspects of the work.

## Ethics Statement

The study was conducted in accordance with the Declaration of Helsinki and approved by the Institutional Review Board (IRB) of the University of Hong Kong and HA Hong Kong West Cluster (UW 23‐510).

## Consent

Patient informed consent was waived in this retrospective study by the Institutional Review Board as it is a retrospective study without active patient recruitment while the data were already de‐identified.

## Conflicts of Interest

The authors declare no conflicts of interest.

## Supporting Information

Additional supporting information can be found online in the Supporting Information section.

## Supporting information


**Supporting Information** The following supporting information is also available. (1) “*Supporting table and figures*” file includes Supporting Table 1: The clinical criteria for financial subsidy under Samaritan Fund in Hong Kong, the date of registration in Hong Kong, and the inclusion date within Samaritan Fund; Supporting Figure S1: Weekly influenza case number from 2014 to 2023; Supporting Figure S2: Weekly AQHI from 2014 to 2023. (2) “*STROBE-checklist*” file includes the STROBE Statement—the checklist of items complied with in the observational cohort study.

## Data Availability

All available data are presented in the manuscript, and no additional data will be provided. Data are not available to be shared.
