# Supplementary material for: Potential Impact of Biologic Use on Asthma‐Related Hospitalization: An Epidemiological Study in Hong Kong
Source: Can Respir J. 2026 Jul 15;2026:5535762. doi: 10.1155/carj/5535762 (PMC13370790; doi:10.1155/carj/5535762)

**Supplementary Materials**

**Supplementary Table 1 The clinical criteria for financial subsidy under Samaritan Fund in Hong Kong, the date of registration in Hong Kong and the inclusion date within Samaritan fund**

| **Biologics** | **Clinical criteria for Samaritan Fund application** | **Date of registration in Hong Kong** | **Date of inclusion in Samaritan Fund subsidy programme** |
| --- | --- | --- | --- |
| Omalizumab | Patients with severe persistent allergic IgE mediated asthma as an add-on to optimized standard therapy in patients age 6 or above who need continuous or frequent treatment with oral corticosteroids, defined as at least 4 courses in the previous year | 3^rd^ June 2006 | 11^th^ January 2020 |
| Mepolizumab | Add-on treatment for severe refractory eosinophilic asthma in adult patients with blood eosinophil count of at least 150 cell/microliter at baseline and at least exacerbations in the past year | 29^th^ March 2017 | 11^th^ January 2020 |
| Benralizumab | Add-on treatment for severe refractory eosinophilic asthma in adult patients with blood eosinophil count ≥ 150 cell/microliter at baseline and ≥2 exacerbations in the past year | 14^th^ December 2018 | 29^th^ December 2020 |
| Dupilumab | Add-on treatment for severe asthma patients aged of at least 12 years with type 2 inflammation (blood eosinophil count ≥ 150 cells/microliter and/or fractional exhaled nitric oxide ≥ 25 parts per billion) who are inadequately controlled (≥2 exacerbations in the past one year) on high dose inhaled corticosteroid plus another drug for maintenance treatment | 30^th^ October 2018 | 28^th^ May 2020 |

**Supplementary Figure**

**Supplementary Figure S1 Weekly influenza case number from 2014 to 2023**

**
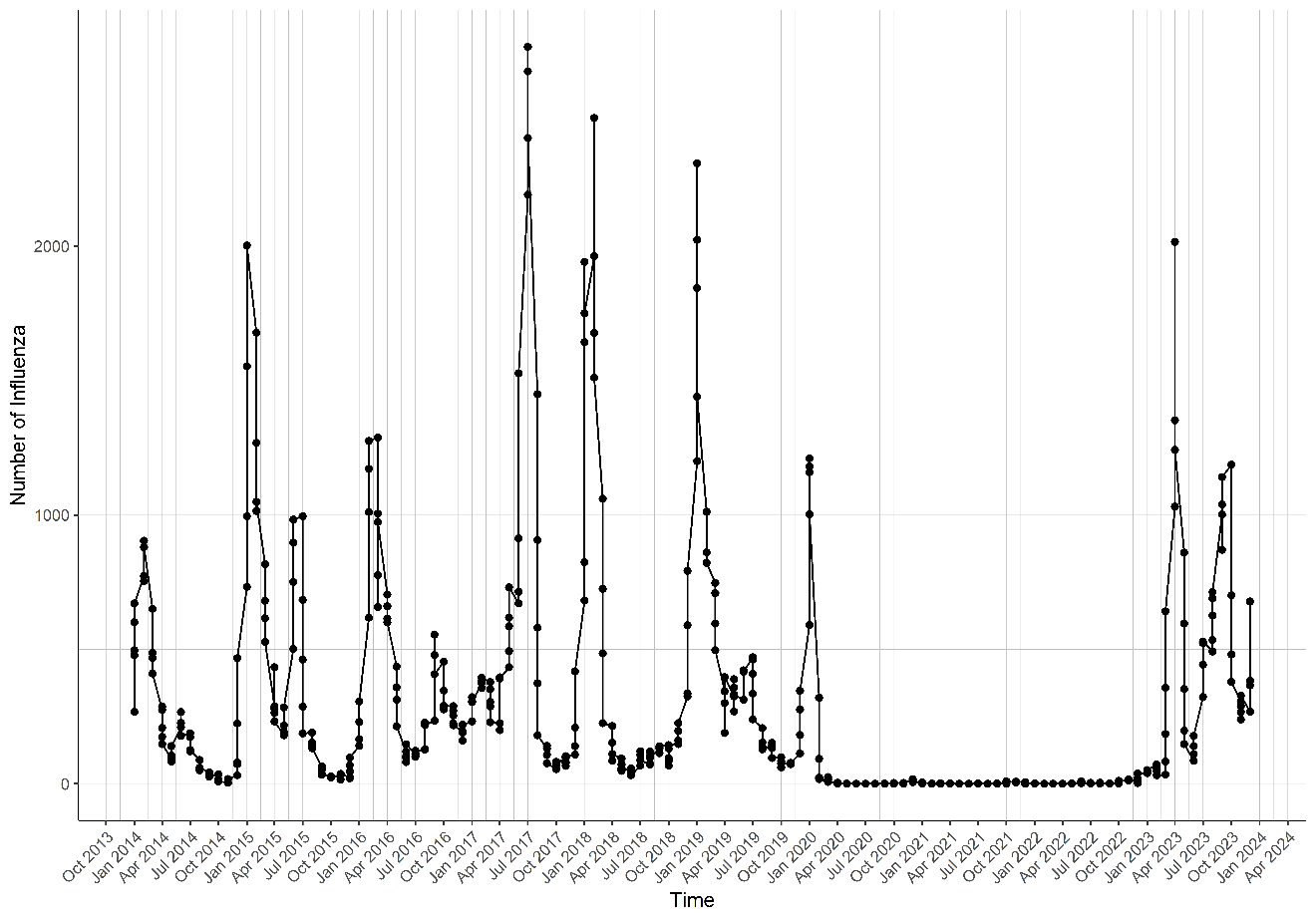
**

**Supplementary Figure S2 Weekly AQHI from 2014 to 2023**


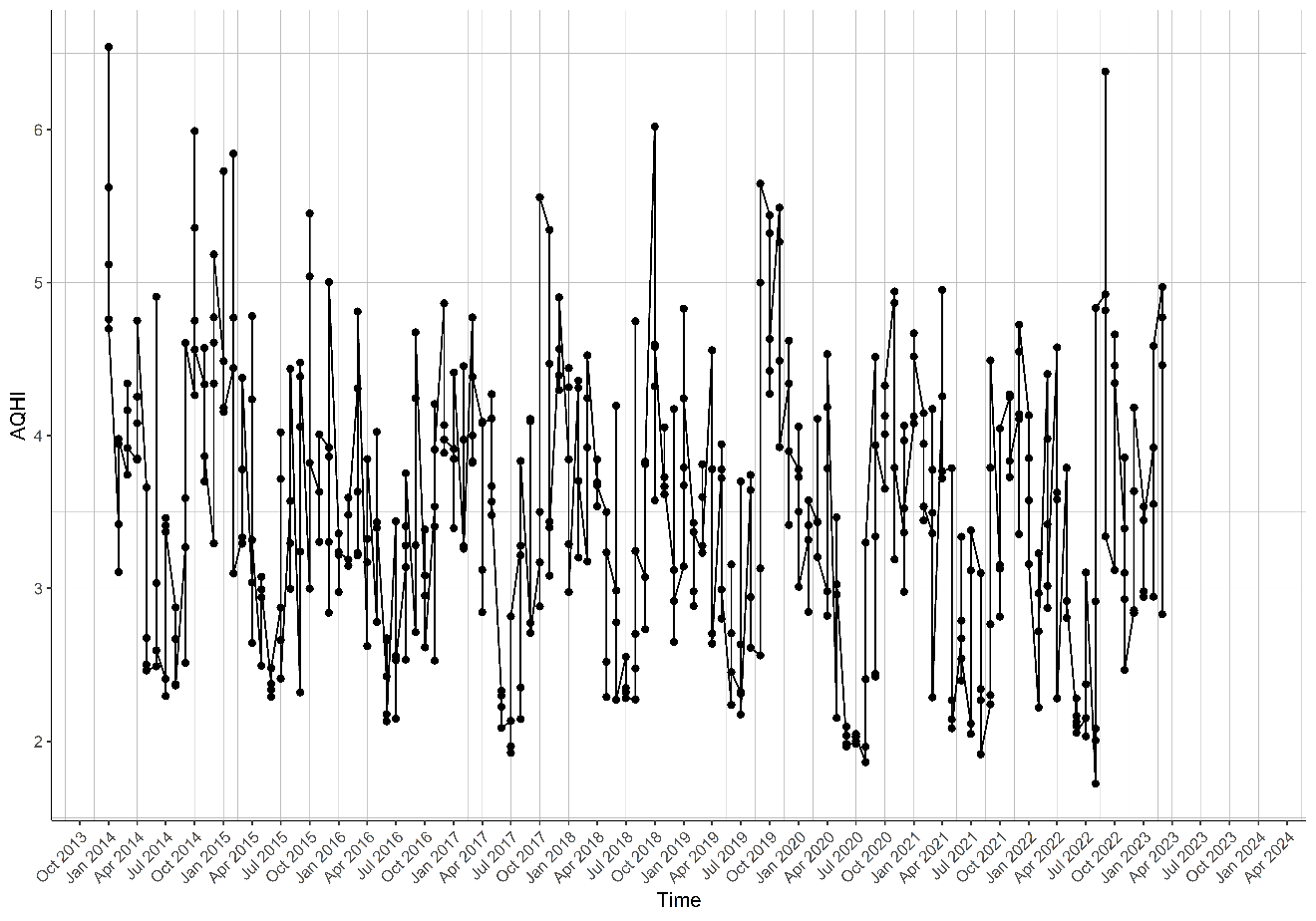

Supplement: Supplementary file 1 — Supporting Information The following supporting information is also available. (1) “Supporting table and figures” file includes Supporting Table 1: The clinical criteria for financial subsidy under Samaritan Fund in Hong Kong, the date of registration in Hong Kong, and the inclusion date within Samaritan Fund; Supporting Figure S1: Weekly influenza case number from 2014 to 2023; Supporting Figure S2: Weekly AQHI from 2014 to 2023. (2) “STROBE-checklist” file includes the STROBE Statement—the checklist of items complied with in the observational cohort study. [file CARJ-2026-5535762-s001.zip › Supplementary table and figure.docx]
